# Adolescents Concerned about Climate Change: A Hermeneutic Study

**DOI:** 10.3390/ijerph20227063

**Published:** 2023-11-15

**Authors:** Kailie Drumm, Roxanne Vandermause

**Affiliations:** 1 Nursing Program, Lower Columbia College, Longview, WA 98632, USA; 2 Omaha Division, College of Nursing, University of Nebraska Medical Center, Omaha, NE 68198, USA; rvandermause@unmc.edu

**Keywords:** climate change, adolescents, climate change worry, climate change anxiety, climate change concern

## Abstract

Climate change is a public health threat on a global scale. Over the last two decades, research has uncovered the myriad health effects of climate change and its associated costs. The literature is also beginning to show the direct and indirect effects of climate change to be an indicator of increased adverse mental health outcomes including excessive worry, anxiety, grief, and post-traumatic stress disorder. The development of scales to measure some of these effects in adult populations has shown the critical need to understand the various ways climate change affects mental well-being in adolescent populations who are at a critical juncture in psychological development. The purposes of this study were to understand the lived experience of adolescents who are concerned about climate change and uncover the meaning of climate change concern for adolescents as informed by emerging patterns. This study utilized Hermeneutic Phenomenology as a philosophical foundation and methodological approach for data retrieval and analysis. An interview-based approach with a purposeful sample (n = 11, aged 12–17 years) revealed the multi-layered elements of climate change concern and its effects. Three patterns emerged: Climate Change as a Temporal Threat and Pressure, Awareness and Concern as a Continuum, and Experiencing Concern and Making Meaning. These findings may now inform interdisciplinary knowledge on upstream mitigation efforts and the promotion of positive outcomes relating to climate change. The need for focused educational attention to adolescent behaviors and concerns is explicated and exemplified.

## 1. Introduction

The threats posed by a changing climate range from physiological to existential. In 2021, the Sixth Assessment Report by the International Panel on Climate Change amassed worldwide research from multiple disciplines and delineated actual and potential threats of a warming climate [1]. Direct consequences of a changing climate include sharply rising mean global temperatures with associated drought in some areas and an increase in rain and flooding in others, glacial degradation leading to increasing and warming sea levels, increased prevalence of wildfires, and loss of sacred and indigenous lands [1,2].

A vast array of health effects of climate change have been reported [1,2,3,4,5,6]. These potential health outcomes include direct and indirect results of the changing climate and its effects on the planet. Indirect examples include increased vector-borne diseases related to the increase in reproduction and habitat areas for the vector due to warming and flooding [4]. Cardiac events have also increased during intense and prolonged heat waves [2]. Still more indirect effects of climate change include displacement due to natural disasters and associated negative health outcomes linked to food and water insecurity [5]. Socioeconomic status and geographical location are now known to be critical determinants of health in the climate change setting as well. For example, underserved and rural populations have decreased access to critical care during and after natural disasters [1]. Drought can cause both a shortage of agricultural production and jobs as well as a decreased food supply in many regions [1]. Underdeveloped countries and populations belonging to underserved and impoverished areas bear the largest burden of these changes.

Direct health effects of climate change include trauma sustained after a natural disaster, increased heat exposure, and progressively widespread wildfire seasons which pose health risks including inhalation of particulate matter and other harmful components of wildfire smoke [7,8,9,10,11].

Physiological factors place children and adolescents at increased risk of suffering the adverse effects of climate change, in part due to their immature physiology and smaller body mass. For example, the rise of viral and bacterial infections due to increased temperatures and, with that, increased vector-borne illnesses affect children and adolescents disproportionately as they have underdeveloped immune systems [3,4]. Thermoregulatory issues may also arise in children and adolescents due to smaller body mass. Additionally, this population is at risk for adverse effects of air pollution which increase the prevalence of respiratory illnesses and asthma exacerbations [5]. A comprehensive list of actual and potential direct and indirect adverse health outcomes as a result of climate change is beyond the scope of this paper; however, recent studies have uncovered a sharp increase in data supporting the need to understand the mental health effects of climate change in addition to physical outcomes. These studies focused primarily on adult populations, with outcomes including grief [12], anxiety [13], and post-traumatic stress disorder [14].

Children and adolescents are a vulnerable population for several reasons. Defined in healthcare as a minority group, vulnerable populations are those who also rely on others for care including older persons and children, those who are under- or non-insured, and those who belong to disadvantaged groups. Children and adolescents may belong to one or more of these groups simultaneously [15]. Adolescents who live in underdeveloped countries or are members of indigenous populations may also experience increased vulnerability to all health or existential threats, including climate change [16].

Adolescents, defined by Jean Piaget as those aged 11 and older [17], are at a critical juncture in their physiological and psychological development. This stage of development is characterized by moral and cognitive functioning and growth [17,18] with external factors including home life and country economics affecting this population well-being. The foundation of research describing the physiological effects of climate change on this population has been laid. However, there exists a gap in research understanding the psychological effects of climate change on adolescents. Research is beginning to emerge that shows mental health outcomes post disaster in children and adolescents [14]; however, little is known about the indirect effect of climate change and its existential effect on this population. This important developmental stage stresses that we better understand what it is like to be an adolescent during the climate crisis. Various groups have published statements demanding more research into the holistic effects of climate change on adolescents, including the American Academy of Pediatrics [3] and the National Association of Pediatric Nurse Practitioners [19]. Given the research that delineates the burgeoning climate-related mental health crises in adults, it is imperative to better understand the adolescent experience as well. These adult-based studies, while valuable, infer mental health outcomes pre-analysis such as anxiety [13,20] and worry [21]. In order to understand why adolescents see climate change as a threat and what forms of coping and interventions may be effective, we must first understand how they experience what it means to be concerned, and how they experience that concern.

Beginning-stage research is uncovering that climate change concern in adolescents can be experienced in various forms, including grief, anger, powerlessness, and frustration [22]. Moreover, research shows that climate change can affect mental health in direct and indirect ways, including physical threats and existential threats that can be immediate or distal [23]. Van Nieuwenhuizen et al. [24] called out the importance of a more robust understanding of the causal inference of various threats due to climate change on mental health. The authors posit that future development of any clinical interventions must be contextually relevant and rooted in an understanding of the holistic nature of climate change effect on adolescents [24].

The purpose of this study was to explore and understand the lived experience of adolescents who are concerned about climate change.

## 2. Methods

Nurses have increasingly utilized interpretive research methods to understand an experience of a phenomenon rather than solely describe it [25]. In keeping with the tradition of nursing philosophy, this type of approach places experience as a key element in the formation of the foundation of knowledge for the discipline. Hermeneutic phenomenology provides a lens to uncover the contextual positioning of the experience and experiencer. For example, the nurse using this approach can understand the meaning of the experience as it relates to the world in which the individual is experiencing the phenomenon, such as demographic and psychosocial factors, which are important aspects of patient-centered care and nursing research.

Phenomenology, as a philosophical approach and a way of thinking, is concerned with the line of questioning associated with experience. The experience of living with concern about climate change must be understood to determine appropriate measures to promote positive outcomes. This study aimed to (1) understand the lived experience of adolescents concerned about climate change and (2) uncover the meaning of climate change concern for adolescents as informed by emerging patterns through the utilization of a holistic nursing approach to interpret findings. The study protocol was reviewed by Washington State University Office of Research Assurance.

### 2.1. Hermeneutic Phenomenology

A hermeneutic phenomenological approach was used in this study to interpret “what it means to be” an adolescent concerned with climate change. The design, data collection, and data analysis were grounded in philosophical hermeneutics [26], providing a distinctive and deeply contextual phenomenological view that resulted in description and interpretation, leading to a common understanding of the phenomenon.

### 2.2. Sample and Recruitment

Participants were recruited via a link distributed by nurses from the Alliance of Nurses for Healthy Environments (ANHE). This allowed for snowball sampling until the desired number of participants was achieved (desired *n* = 10–15; actual *n* = 11).

Inclusion criteria were: (1) adolescents aged 11 to 17, (2) English-speaking, (3) residing in the United States and (4) identified as being concerned about climate change. It was not the intent of this study to paint a picture of the overall perception of climate change by all adolescents, but to understand the nature of concern as it relates to being; therefore, the sample elicited included those who identified as being concerned. For a hermeneutic phenomenological study, a sample size of 10 to 15 was targeted to achieve an appropriate understanding of themes and patterns [26]. Hermeneutic phenomenology does not delineate a specific number of data sources to be an effective method (such as a power analysis). Instead, sufficient data collection commenced when the analysis team no longer found novel patterns and/or themes in the data necessary to describe and represent the phenomenon [26].

### 2.3. Data Collection

Interviews took place between September 2021 and August 2022. Analysis of transcripts occurred simultaneously and through October 2022.

This study had two phases: pre-interview interactions with the participants and the interview itself. Pre-interview interactions included sending consent and assent forms to participants via email. The interviews took place at a mutually agreed-upon time via Zoom. This allowed for the recruitment of participants from various areas of the country.

The PI (KD) was the sole interviewer. At the beginning of the interview, questions about the study and forms were solicited from the parent or guardian and the participants by the PI and answered. If present, the parent or guardian was then asked to leave the interview space for privacy. A brief demographic questionnaire was then administered to determine (1) age, (2) preferred gender, (3) ethnicity, and (4) rural, urban, or suburban location. The participants were then asked to provide a pseudonym of their choice, which was recorded in the transcripts.

All interviews then followed with the open-ended question: “Please tell me what it means to you to be concerned about climate change”. To uncover a more detailed understanding of the lived experience of these individuals, this open-ended question was followed by probing questions to gain clarity, such as “Please tell me more about that”, “Can you explain that further” or “What does that mean to you?”. This technique is in keeping with the methodology’s philosophical tenets of hermeneutics [27,28]. Each interview lasted approximately sixty minutes via Zoom and was audio-recorded using a separate recorder. Video was not recorded. Immediately following the interview, field notes were taken by the PI to capture aspects of the interview that audio could not, such as body language, interpretations of the interview process, and reflective practices. Field notes were analyzed by the interpretive team alongside the transcripts and the interpretive accounts as part of a multi-level text [29] that comprised the database.

Resources were made available to the participants in the event they exhibited signs of anxiety, depression, or other emotional trauma. The resources were also made readily available on the recruitment website that was sent to participants. All correspondence that contained participant identifying information was stored in a password-protected computer utilizing the secure server provided by Washington State University and deleted afterward. All other study materials used de-identified information and pseudonyms and were stored in a locked office.

### 2.4. Data Analysis

A hermeneutic analytical approach [26,30] was utilized to analyze the de-identified interview transcripts. Members that formed the interpretive team included the PI and four doctorate-prepared nurses, including a climate change expert with extensive phenomenological experience, three hermeneutic phenomenological experts, and a PhD-prepared pediatric nurse practitioner with research experience in qualitative and quantitative methods. A major tenet of hermeneutic phenomenology is rooted in the philosophy that the researcher should not bracket or deny their experiences and presuppositions, but rather utilize them in the data, being consciously and continuously aware of them [26].

The study design utilized the guiding principles of the philosophy of hermeneutic phenomenology for both the interview process and the data analysis. This study aimed to uncover the participant’s experience of the phenomenon and how it relates to their being-in-the-world. Following an iterative analysis of each transcript by all analysts, emerging patterns and contributing interpretations were collected and summarized.

## 3. Results

Eleven participant stories were included in the analysis. Participants represented 12–17 inclusive years and belonged to varying demographic groups, including ethnic minority groups and rural areas (see Table 1).

If the subject of mental health was broached by participants, they were asked about the effect of climate change on their mental well-being. Almost all participants denied an overt or pervasive effect of climate change on their mental health but did describe moments of anxiety, worry, fear, frustration, and guilt. Stories of depression and anxiety were shared in some interviews. Participants attempted to make a clear distinction between the temporality of those emotions, such as in moments when presented with photos of extinct animals (acute emotion) versus the pervasive and chronic nature of those emotions on overall mental health; most identified as falling into the former group. Participants generally fell into two categories: those who used uncomfortable emotions as motivation to participate in climate action and those who were still growing into an awareness of climate change and deciding what to do about it.

### 3.1. Patterns and Themes

The participants’ stories uncovered a dynamic and evolving experience for the adolescents as they grappled with understanding climate change. This was a multi-faceted process that involved learning about climate from a scientific perspective and then translating that into a personal story in which participants sought to understand how climate change affects them. Three patterns came into view across all texts: Climate Change as a Temporal Threat and Pressure, Awareness and Concern as a Continuum, and Experiencing Concern and Making Meaning. Encompassed within those patterns are themes that further represent the experience (see Table 2).

#### 3.1.1. Pattern 1: Climate Change as a Temporal Threat and Pressure

Climate Change as a Temporal Threat and Pressure emerged as an overarching pattern. Participants openly grappled with the dichotomous understanding of climate and the inference of “change”. They described how “climate change” is an issue that affects them in their current daily living but also forces them to consider their future and how it could be altered. To add to this conundrum, many shared that the knowledge of previous generational experiences affected their current feelings and perceptions of the changing world, with associated feelings of grief and frustration.

##### Theme 1: Climate Change: What do I see?

Participant experiences of climate were described both from a firsthand, experiential perspective and an abstract future-oriented point of view. Adolescents identified how the climate affects them personally, through stories of summers impacted by increased wildfire smoke and drought along with decreased length of winters. They also experienced climate from afar, through the news of natural disasters, reports of projected increases in these disasters, and information related to how the future will differ from the past because of climate change. When asked about his experience of climate concern and what it means to him, Kaleb immediately described the tangible and concrete effects of climate on his ability to enjoy his favorite activities:

I guess the smoke prevents me from going outside. So that affects how much outdoor time I can get. I can’t go in the water as much because it like, the water areas will be pretty low with how hot it’s been getting especially this summer (Kaleb)... and I like to spend a lot of time in the winter so that will affect me. ‘Cause in the winter I spend a lot of time outside like snowboarding and sledding and stuff (Kaleb).

Delusional, who often checks headlines daily for news of climate and natural disasters, began his story by describing the effects of climate that he sees:

I mean, temperatures are consistently rising and like especially in my area we have had a tornado in recent years, and we just had one a few weeks ago... That the northeast doesn’t really get those, and I was like, wow, I mean, severe weather. That’s a thing that’s supposed to be coming with climate change and I’m like that’s uh, not really supposed to be happening (Delusional).

Participants fell onto a continuum of climate change awareness and understanding of the effects of a changing climate. Older teens expressed more macro-level considerations. That is, all participants came to understand climate as it relates to their being-in-the-world; however, younger participants shared experiences and stories of climate change and their concerns as it affects them in their personal space while older participants, or those who had known of climate change longer, grew to expand their understanding of climate effects to include the greater world. As the participants described climate change, its effects, and their concern, a deeper theme emerged: *Living in the Now and for the Future*.

##### Theme 2: Living in the Now and for the Future

Participants continually expressed concern and the effects of that concern as they arise in the present, while also continually reshaping their expectations of the future. Aaron described this juxtaposition as follows:

This is a fight that we’re gonna be fighting maybe for the rest of our lives... I was on my run this morning with my buddies, and it was like- and I was talking about- I wonder what it would be like to live like the childhood kind of that my parents did. Right? Where you didn’t have like, you know, this- this, um- this like terrible thing like looming over your head all the time. And you didn’t have to—you didn’t have to like pick up the sword, right, and- and go fight for your future and for your children’s future and for, you know, your family’s future (Aaron).

This experience portrays concern for the present, and how that concern affects Aaron’s sense of his future. All participants spoke about climate change from a temporal perspective. There is an everydayness to climate change, whether they are directly affected by it or not. Climate change, for them, is a way of being in their world. It is a part of their daily living, whether they express overt concern or not. The concern may be rooted in a burden of pressure to do something about climate change presently or worry over the loss of a viable and fulfilling future.

In recounting this way of thinking about climate change, Bob clearly described the overlapping of living in the now and for the future:

And I think, one of the questions about, you know, what does it mean to be concerned about climate change is how to sort of grapple with that mindset of this is an ongoing thing, so I’m going to adapt with it while also doing everything I can to prevent it at the same time... But I think a question that I still haven’t solved is how do I—how do I balance these two mindsets of overcoming this and like accommodating for this, integrating this into my like everyday life while also doing everything I can to prevent it in the future? (Bob).

There is some overlap between the thought of one’s future and other themes such as generational burden and mental and emotional consequences, which is further explored across the interviews in *Pattern 2: Awareness and Concern as a Continuum; Theme 2: Climate Change as a Weight and Burden.*

Not every participant identified as being worried about their future. When sharing how she sees her future and whether her plans were affected by climate change, Robin stated the following:

No. Mainly just where I want to live. I know I want to live somewhere up north ‘cause I like colder weather. But that’s pretty much it. Like now my future is which college I’m going to apply to or which classes I’m going to get next year (Robin).

Climate change, experienced by adolescents as the current climate, is time-bound. The temporality of climate is a perceived threat to the adolescents. There is a burden and pressure to address climate by acknowledging it and attempting to live presently while also worrying about the future. For participants, climate change encompasses the past, the present, and the future. How they became aware of the climate situation was experienced on a continuum, usually in tandem with the developmental continuum [17,18], though accelerated, and is described in the next overarching pattern.

#### 3.1.2. Pattern 2: Awareness and Concern as a Continuum

Participants could point to a time when they first learned about climate change. Usually, this was during a middle school science course, and in some participant cases, this semi-removed and limited knowledge of climate change remains the bulk of their experience of the phenomenon. Participants reflected on what it meant to them to be concerned about climate change and uncovered varied levels of understanding and action related to the phenomenon. Throughout the interviews emerged a spectrum of sorts. The stories of climate change presented themselves on this continuum. Younger participants or those who were just learning about climate change were beginning to grow into an awareness of the issue and grappling with emotions related to that awareness. Other participants who were older or had known about climate change for longer were further along the continuum and often expressed more action-oriented thoughts regarding climate change. These findings are further described in *Theme 1: Understanding Climate Change: Aware to Active.*

##### Theme 1: Understanding Climate Change: Aware to Active

A key finding in this study was how participants learn about climate change. Many participants shared that the most influential method of learning about climate change was through digital media in the form of television or social media. The participants often used these sources to connect the abstract and sometimes unseen crises of climate change and make sense of it in their own lives.

Aaron shared that he was lucky to learn about climate change in his seventh grade earth science course, as his peers not twenty miles away did not have the same opportunity. He disclosed an example of the awareness continuum, from beginning awareness to an emergent sense of action.

And you know what really struck me what that this was something that was just so existential and so—like such a big issue. And I think that struck like really all of my classmates, and yet it still wasn’t really something that we talked about… and so it was just—it just felt very like isolating and scary. And so, for like two years, I really kind of internalized that, and like, you know, I stopped buying new clothes. Which I still don’t buy new clothes. But, I like started recycling and learned about our city’s recycling plan and everything. But then, I slowly like began to realize that like this issue is so systemic, and we can’t recycle our way out of climate change (Aaron).

Bob shared the moment he learned about climate change that echoes child and adolescent developmental stages [17,18] with regard to expanding his worldview and a growing understanding of his place in the world. He described, in his sharing, the moment of awareness, the expanding into that awareness and how that affects one’s being-in-the-world, and his transition from awareness to action:

And I think, um, it was a combination of factors for me. I—I think one—the biggest part for me is probably—it was probably around seventh or eighth grade when I started, you know, paying attention to what’s going on in the world more. Because, of course, when I was like in elementary school, and even like sixth grade—maybe even mostly seventh grade—you’re in this like bubble, you know? My—my life is comprised of school and, you know, coming home and doing homework—whatever. And then at a certain age, probably sometime in middle school, I started thinking, you know, wait a minute. Like there’s actual stuff going on in the world outside of my little bubble… And in some point in middle school, I started reading these headlines. There’s one I remember in particular…Like an iceberg, the size of Delaware has just, you know, gone and broken off…And I think that- that- headlines like those started to remind me maybe- wait a minute. Maybe- maybe this isn’t going to be solved by the time I’m (laughter)—like maybe- maybe- I need to do something about this… (Bob).

As awareness grew, participants shared varying experiences of how that growing awareness affected their mental and emotional well-being, explored in the next theme.

##### Theme 2: Climate Change as a Weight and Burden

Threaded throughout stories of awareness and action along the continuum were experiences of mental and emotional consequences related to climate change. While most participants denied climate change concern being a chronic negative mental health antecedent, some shared stories of fear, anxiety, frustration, and hope.

I could say there’s more—there’s also periodic feelings of like sadness or like depression because like the weight of the situation sits in especially with the extinction of species and the melting of the polar ice caps. And then there’s…hmm… feelings of like, especially when you read news about things that are actually helping like I know this isn’t recent but when countries banned a chemical that was causing the whole in the ozone layer in the arctics, that—when I read about that, even a few years after it happened, that really helped me get hope and gave me happiness. Because with that article it also said that the ozone was healing. So, I feel like there’s a mix of emotions that come with the topic (Delusional, l. pp. 150–158).

Others shared that the climate action they were passionate about and found meaning in came with a risk of burnout and its own side effects. When describing their experiences of climate change action and advocacy, Eleven said:

If feels less like we can just relax and be kinda carefree. But there’s just like—if we want to—if we’re worrying about our future, then it just feels kinda wrong to just be—to just relax and say, oh, we’re teenagers and carefree and wait a while. Because the clock feels like it’s just ticking. (Eleven).

Throughout the interviews were examples of how varying levels of awareness held a common thread of generational pressure. Stef described her experience with the burden of climate change on her generation’s shoulders as the hope for change:

I think people see it as younger generations have different ideas and there is some kind of pressure on like, our generation, like on our shoulders because the older generation—like older people- expect us to be the change (Stef).

The overarching continuum-based pattern and its themes presented the nature of awareness of climate change. Additionally, the study uncovered the multi-layered effects climate change may have on emotional and mental health, whether acute or chronic, with most participants sharing that acute emotions were most prevalent, though still at times distressing. This was shown from the lens of concern as it related to tangible or abstract ideas of climate change, generational pressure, and strain from climate action and advocacy. The following theme, *Theme 3: The Middle Way*, continues with these temporal ideas and uncovers the ways that adolescents shift their expectations about their future in light of climate change.

##### Theme 3: The Middle Way

As the participants learned about climate change, their expectations of the future shifted. What begins as a question about viability becomes an understanding of the changing nature of their future. Younger participants articulated this by asking whether their future jobs would even exist when they became adults. Older participants generally shifted from this line of questioning to instead ask questions such as “What job will be available, and can I enjoy that?”. This is an example of sense-making and meaning-focused coping as explored by M. Ojala [31]. This theme disclosed itself as finding a “middle way”, or a paradigm shift in how the participants see their futures in light of climate change. Often, they described this shift as a way to accept the inevitable: their future looks drastically different than what older generations experienced, and yet, they could still have a full and happy life. Finding this “middle way” was most often facilitated with meaning-focused action and advocacy. Eleven described this resoluteness:

So, when that’s kind of a roadblock, I have to—sometimes I just think about, okay, so if climate change does get worse, how can my future still be my future? And maybe it looks different—but will it still be okay? Like I can still have a family. We can still do things that we like. It’ll just need to be adjusted… And I just think about how there could be more of a middle ground. Where it’s not gonna be the same as it is today or twenty years ago, but maybe we can do enough so it’s not as horrible as a Code Red for humanity (Eleven).

The following pattern, *Pattern 3: Experiencing Concern and Making Meaning*, further represents how that awareness and concern manifests as important to the participants and how they make meaning out of their experience. Additionally, further exploration into how the middle way, and action and advocacy, affect agency in one’s being-in-the-world is explored.

#### 3.1.3. Experiencing Concern and Making Meaning

The study participants represented a dynamic and varied understanding of climate change and diverse levels of concern and advocacy and action regarding the issue. What was consistent, however, was that participants felt that action, in any form, provides a positive outlet for these adolescents to understand, interact with, and make sense of climate change.

##### Theme 1: Agency and Release through Action

This study uncovered many ways one can act as a form of advocacy or coping. One of the key findings showed that talking about climate change is a form of action. This action offered many participants a sense of agency or relief from the burden of their concern and feeling isolated. Whether or not an adolescent identified as being heavily engaged in climate activism and incredibly concerned about the crisis, all participants spoke to the power of talking about the issue as a form of release or action.

Jane and Delusional both pointed out their frustration at the apparent lack of power and agency they may have over the circumstances given the overwhelming nature of the issue. While some participants struggled past this and found a sense of agency through their actions, participants such as Jane and Delusional still struggle to see that their generation could accomplish much in the face of climate change. Both participants, however, found some sense of agency through discussing climate change with supportive others:

Jane: And so, it’s sad that some people don’t care, and others that do care can’t really do much about it…Sometimes our teacher will bring up stuff. Sometimes she’ll ask us how we feel about it [climate change], which is definitely very helpful (Jane).

Delusional: I don’t feel that I personally can at this age, especially, do a lot of change and create a lot of change, but I think that having these talks with my friends help… [It’s] a way of securing ourselves and helping cope with what’s happening and feeling more helpful in the situation (Delusional).

Several participants described a common experience of transitioning from learning about climate change and the isolation of that knowledge to a sense of agency. Fear, anger, and hopelessness were emotions expressed by these teens when they were initially presented with climate education. There is a common sense of being thrown into a world they inherited. Each of these participants pointed to the time when this isolated feeling lessened, often occurring when a friend, teacher, or community member offered them the opportunity to talk about their feelings or participate in climate advocacy and action. However, an important factor in this discovery included the presentation of opportunities to engage in action of some sort. After his interview, one participant shared that if it were not for his older friends who allowed him the chance of participation in a climate rally, he would not be where he is now. This experience was shared by all participants who were climate active. For those who were just becoming aware of climate change, the desire was focused on seeking out someone to talk to about their emotions and to learn more about the climate situation. In those who were more active in climate advocacy, the regenerative effects of the communities they belong to were a protective mechanism against despair and hopelessness.

## 4. Discussion

The aim of this study was to understand the lived experience of adolescents who are concerned about climate change. Climate change and its effects on various populations is a developing field of inquiry, especially regarding adolescent populations; therefore, a holistic methodological approach was necessary to answer this novel question. At the end of every interview, participants were asked about the best way for nurses and other adults in their life to support them. Their answers are threaded throughout the domains of this section.

### 4.1. Education

#### 4.1.1. Adolescent Education

All participants identified a gap in their education as it relates to climate change, which was often a source of frustration. While some participants did receive climate-related education in their earth science courses, this was not universal across all participants. Those who did receive climate-related education in school spoke about the abbreviated nature of the topic, wherein climate change was briefly introduced without any room for discussion afterward.

Adolescents are particularly vulnerable to encountering an alarming article or newsreel that they are unable to adequately fact-check. Some participants wanted a more robust and reliable source of information to be readily available to them. Adolescents see the paradox of having limited education on a topic given so much importance in the outside world. They want to learn about climate change in school where they can ask questions and sort through information with a trusted adult. Similarly, they need discussions on how to critically assess the media and the social debates on the topic.

#### 4.1.2. Nursing Education

Nursing education must reflect the updated 2021 American Association of Colleges of Nursing (AACN) Essentials, such as AACN Essential 3.6b which speaks to the need for climate change in nursing curricula [32]. One of the most concerning comments from a participant in this study was that she did not find her school nurse helpful, and three participants stated that they saw the school nurse as someone for physical ailments but not a resource for socio-emotional support. Future nurses need to know about the vast ways in which climate change can affect our patients, clients, students, and neighborhoods. Similarly, school nurses can begin to advocate for more resources and seek content to use in their practice, including education and training to identify and support those who may be experiencing climate-related emotions.

### 4.2. Policy

The policy implications of this research are wide-ranging and encompass many stakeholders. These include local and federal legislators who aid in curriculum development at public schools via funding sources, local school boards who advocate for and implement curricula and learning standards, and anyone who cares for adolescents. There is an educational disparity between rural and urban education and policy surrounding climate change. Those participants who lived in rural and politically conservative states struggled to find climate-related education and support for implementing initiatives to manage the climate crisis. This was evident in those who attempted to make climate-related clubs at their schools and in those who received no climate education but learned of the crisis from the news and media. These adolescents learn about climate change no matter where they live, but lack regional policies to provide appropriate education surrounding climate change to mitigate confusion and stress-related sequelae.

Some participants also spoke about the barrier they perceived at approaching various adults in their schools regarding climate change. For example, those who wished to begin a garden or chess club at their school were not met with the same barriers as those who wished to start a green club. Some felt that the political nature of climate change was preventing many teachers from volunteering time as advisors for clubs for fear of backlash from school district administrators. Advocacy for the protection of teachers interested in aiding in forming these clubs must occur, beginning with the school board.

### 4.3. Research

This study disclosed the lived experience of climate change concern for adolescents. It provided the framework for various lines of inquiry, not only for nurses but for other disciplines as well.

#### 4.3.1. Nursing Research

Findings from this study brought to the forefront nursing research gaps that were evident prior to this study. Nurses in a research setting should seek to better understand how climate change affects adolescents. This study is a launching point for future work. Further nurse-led research should expand to include adolescents from a broader demographic spectrum to represent various environmental-justice factors. Levels of climate change concern and its effects on teens may be influenced by where they live and the associated burden climate change has on their environment. Additionally, this study specifically targeted adolescents who identify as being concerned about climate change. Future studies should include those who do not, as this may aid in understanding what gaps exist in climate education and uncovering the relationship between factors affecting climate concern.

Longitudinal studies would be beneficial for this line of inquiry. Those who are younger would provide a valuable data set to corroborate study findings in which a natural progression occurred from climate aware to climate active as one ages across the developmental spectrum.

The development of scales is needed to identify adverse mental health outcomes in an upstream fashion and this has indeed already begun [13,21]. However, before this can occur, the elements of concern, worry, or anxiety related to climate change need to be identified. This work could occur in various pockets of high-risk areas such as those regions of the United States that are more prone to climate-related disasters or those areas that are disproportionately affected by environmental justice issues. Study findings also point to the need for further research into what appropriate measures exist or need to be developed to mitigate adverse outcomes and what existing protective factors can be promoted by nurses.

#### 4.3.2. Interdisciplinary Research

Further analysis of the findings as they relate to developmental theories and adolescent education is important. This study in some ways echoed the developmental theories of Piaget [17] and Kohlberg and Hersch [18] wherein adolescents follow a developmental trajectory of awareness regarding the greater world, their place in it, and moral obligations to act on behalf of the conceptual greater good. However, with a changing world that is largely affected by the influence of unfiltered media instantly available, clear and accurate information is elusive. Adolescents are developmentally less able to critically analyze the barrage of information. Some of these participants were taught climate education in school, but almost all spoke about the digital sources that they found on their own as being most fundamental in their understanding of the crisis. This reflects a new way in which students may prefer to learn, as they grasp onto television shows or movies they enjoy and utilize those to make sense of climate change. Educational research and curriculum development should reflect this changing world of information sourcing and learning modalities.

Finally, the heightened level of awareness of the world due to the internet calls for updated developmental theories to guide this new problem. There is an accelerated development that is occurring where younger age groups reflect a more mature understanding of the world and question their place in it at a younger age due to globalization and information availability. Interdisciplinary scholars and practitioners are needed to create learning solutions.

### 4.4. Practice

Findings from this study showed that agency and positive outcomes can occur through action, and that action may occur in the form of information seeking or simply discussion. Any group that is in contact with adolescents should promote and facilitate this outcome. This includes school counselors, teachers, parents, school nurses, mental health professionals, and clinicians.

One of the key findings is related to adolescents’ need for mentors with whom they can share climate-related questions or worries. They looked to their teachers or school counselors and were keenly aware of the “touchy” subject of climate change, and the barriers their teachers and various staff sometimes faced to speak about climate change with their students. This barrier is a potential mental and public health emergency, and no teacher who is approached by a student needing help should turn from this opportunity for fear of punitive action from their superiors. Adolescents in this study gained a sense of belonging and a sense of coherence about climate change when they were part of a group of other individuals who shared their experiences. These opportunities need to be provided for all students to mitigate the stress this cohort expressed.

This study uncovered important findings related to agency building through climate action. Participants who were climate active shared a wide range of action that was inherently person-driven and spoke to their individual interests. For example, one participant was not comfortable with public speaking but was an artist. She chose to utilize her passion for art to support climate action by drawing, painting, and utilizing digital media to create flyers that others would use for climate strikes. Two participants who were very passionate about the outdoors channeled their climate action towards environmental conservation tasks and joined clubs where the focus was on the preservation of sacred or endangered lands. One participant learned the importance of recycling and reusing products to decrease their carbon footprint, so they focused their climate action on repurposing clothing purchased at second-hand stores along with their passion for sewing to make their own clothing. Throughout the stories, participants said that it was important to them to find climate action that spoke to their interests as this could prevent burnout. Again, this echoes the work on M. Ojala [31] wherein meaning-focused coping that reframes an issue and allows for one to find value-based coping is imperative. Opportunities must be presented to adolescents to engage in person-centered climate action that speaks to them and their interests. One may feel overwhelmed when asked to orchestrate a letter to a senator; however, engaging in an activity that is within their locus of control and is comfortable to them can ease the overwhelming nature of where to start that even adults are facing regarding climate action.

### 4.5. Limitations

This analysis was bounded by adolescents who identified as “concerned about climate change”. Their stories were deeply examined but there is likely a breadth of experience over the range of adolescence that was untapped. Further, the developmental trajectory that showed up in the study could not be adequately examined for inference with the distribution of participants in the study.

## 5. Conclusions

This study uncovered the dynamic and multifaceted lived experience of climate concern in adolescents. The main findings include the temporal nature (immediacy) of the experience, the everydayness of climate events and their associated urgency for action, the need for a more robust education effort, and the missed opportunities to provide support and a sense of coherence for adolescents. The physiological and psychological toll of climate change on all populations is becoming a public health crisis, including for the vulnerable population that is adolescents.

Healthcare and social science professionals and practitioners understand the importance of upstream mitigation efforts in preventing adverse public health outcomes [33]. Similarly, factors that define a vulnerable population are understood, which includes adolescents who depend on adults to protect, advocate for, and educate them [15]. Lastly, the importance of a sense of belonging and coherence to mental health is also well known [34,35]. In this study, these concepts are brought together and the relationship between them is illustrated. The findings of this study help to better understand what it means to be an adolescent who is concerned about climate change, which is a current and urgent problem, and what public health professionals can do to promote positive outcomes and alleviate the burden of that concern.

## Figures and Tables

**Table 1:** Demographic Data.

Age Range	Gender Identification	Ethnicity/Race	Area of Living
12–17 years	Male Female Non-Binary	White Asian-American East Indian Hispanic	Rural Urban Suburban

**Table 2:** Patterns and Themes.

Pattern 1: Climate Change as a Temporal Threat and Pressure	Pattern 2: Awareness and Concern as a Continuum	Pattern 3: Experiencing Concern and Making Meaning
Theme 1: Climate Change: What do I See?	Theme 1: Understanding Climate Change: From Aware to Active	Theme 1: Agency and Release through Action
Theme 2: Living in the Now and For the Future	Theme 2: Climate Change as a Weight and Burden	

## Data Availability

The data presented in this study are available on request from the corresponding author. The data are not publicly available due to privacy restrictions.
